# Association between serum cotinine and volatile organic compounds (VOCs) in adults living with HIV, HBV, or HCV (NHANES 2005–2018)

**DOI:** 10.1038/s41598-022-26420-7

**Published:** 2022-12-16

**Authors:** Jie Yang, Hao Zhang, Jin-Long Lin, Jing Liu, Xiao-Wen Jiang, Lei Peng

**Affiliations:** 1 Major Infectious Diseases Management Department, Public Health Clinical Center of Chengdu, Chengdu, 610066 China; 2 Department of Social Medicine and Health Education, School of Public Health, Peking University, Beijing, 100191 China; 3 School of Marxism, Tsinghua University, Beijing, 100084 China; 4 Institute of Population Research, Peking University, Beijing, 100871 China; 5 People Liberation Army Haidian District 17th Retired Cadres Rest Home, Beijing, 100143 China; 6 Department of Epidemiology, School of Clinical Oncology, Peking University, Beijing, 100142 China

**Keywords:** Endocrinology, Health care, Risk factors, Chemistry

## Abstract

Although people all know that nicotine in tobacco smoke is the key to cause health damage, they ignore the synergistic effect of a large number of Volatile Organic Compounds (VOCs) produced by incomplete tobacco combustion on nicotine or cotinine metabolism. Our aim is to investigate the association between serum VOCs and cotinine in smokers infected with HIV, HBV or HCV. National Health and Nutrition Examination Survey (NHANES 2005–2018) database, including 13,652 nationally representative subjects’ sociodemographic characteristics and serological indicators, was used in this study. Smokers living with human immunodeficiency virus (HIV), hepatitis B virus (HBV) or hepatitis C virus (HCV) were compared to non-infected population. The correlation between VOCs and cotinine as well as the effects of VOCs on cotinine metabolism were analyzed by Spearman correlation analysis and multivariable logistic regression analysis, respectively. Among HIV, HBV, or HCV infected smokers with the largest exposure dose to tobacco, the intensity of the association between VOCs and cotinine was the strongest. The results of multivariable binary logistic regression showed that high concentrations of 1,2-Dichlorobenzene (OR:1.036, CI:1.009–1.124), Benzene (OR:1.478, CI:1.036–2.292), Carbon Tetrachloride (OR:1.576, CI:1.275–2.085) and 2,5-Dimethylfuran (OR:1.091, CI:1.030–1.157) in blood might be independent risk factors leading to the increase of serum metabolite cotinine in smokers.

## Introduction

Cotinine has been proved to be an exposure biomarker for monitoring tobacco use, which is most often determined in urine, serum and saliva, has longer half-life (16-h) than its precursor nicotine (2-h), and reflects the amount of smoking and metabolic status of the body^1,2^. The serum cotinine level increased in a dose-dependent manner with the increase of tobacco use or exposure^3^. A study showed that smokers exposed to the same amount of tobacco were more likely to become addicted if the body absorbed more nicotine, resulting in the decline of smokers' ability to quit smoking^4^. Exposure to nicotine, a precursor of cotinine in tobacco, could lead to green tobacco sickness and DNA damage^5^. Cotinine could also affect the reproductive function of men and women and increase the risk of cancer in offspring^6^.

Tobacco could produce a large number of toxic substances under incomplete combustion, and the most typical was VOCs^7,8^. Studies have shown that benzene was one of the carcinogenic and popular toxins in cigarette smoke, which belonged to VOCs and was related to smokers' leukemia^9,10^. One study measured the individual cotinine exposure of 72 smokers, and then calculated the benzene exposure. Compared with non-smokers, smokers have higher benzene exposure^11^. In addition, researchers also found other VOCs types in tobacco smoke, such as monoaromatic hydrocarbons (including ethylbenzene, toluene, styrene and xylene), and these might pose a certain risk to health^12,13^. Smoking, a social behavior, would lead to the absorption of nicotine and VOCs. At the same time, VOCs absorbed into the blood could inhibit the metabolism of cotinine by cytochrome P450^14–17^. It showed that when the body absorbed the above two at the same time, there could be a synergistic effect. Therefore, The quantitative measurement and joint analysis of VOCs and cotinine or nicotine are essential, because all of these toxins can circulate to important organs and tissues with the blood^18^.

Obviously, different VOCs have the same status of harm to human health, but the relationship between VOCs and cotinine is also indivisible, because their joint harm to health may be greater than that caused by them alone. For example, one study found that 2,5-dimethylfuran in VOCs could be used as a smoking monitoring biomarker comparable to cotinine^19^. In addition, Pauwels C's study pointed out the correlation between VOCs and cotinine, but did not show the one-way effect of VOCs on the metabolic level of cotinine^20^. Therefore, now it is urgent to use more representative samples to identify the synergistic effect of VOCs content in bleeding fluid on cotinine absorption and metabolism levels, because a few small sample studies only confirmed the correlation between them, rather than the causal relationship^18,21^. The key subjects we selected were smokers living with human immunodeficiency virus (HIV), hepatitis B virus (HBV) or hepatitis C virus (HCV), whose tobacco exposure was much higher than that of ordinary smokers, which was an effective and special place for us to solve this research question.

Generally speaking, the smoking rate of some patients with blood infectious diseases was often higher than that of the general population. According to a study report based on the United States integrated health system, people living with HIV were more likely to smoke and more vulnerable to the harmful effects of smoking than people without HIV^22^. Another study showed that hepatitis patients in the United States were mainly drug users, non-Hispanic blacks, the poor, people with low education and people with mental health disorders^23^. The typical feature of these groups was the high smoking rate^24^. Therefore, in this study, smokers living with HIV, HBV and HCV were studied as a group of high tobacco exposure people, in order to find out the more real association between serum VOCs and cotinine metabolism than the general smokers, compared with non-infected smokers.

## Methods

### Study sample selection

**Figure 1:**
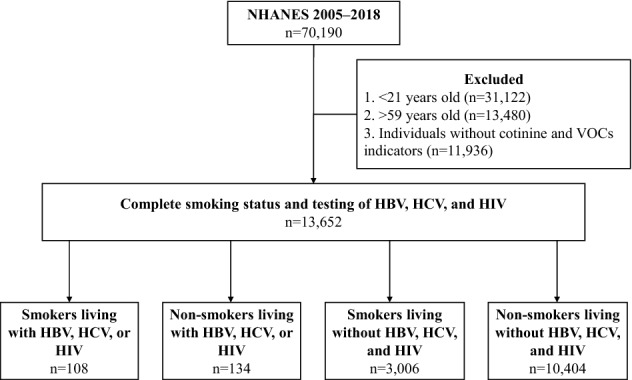
Flow chart of research subjects selection.

The materials used in this study were 14-year long-term follow-up survey data from Centers for Disease Control and Prevention (CDC) in the United States, which were a biennial survey conducted by the National Center for Health Statistics (NCHS). The 2005–2018 National Health and Nutrition Examination Survey (NHANES) data sets required for the analysis were downloaded from the NHANES website and combined using SAS software version 9.4. A total of 13,652 eligible subjects aged 21–59 was included in this study (Fig. 1). They had complete general sociodemographic information, as well as serological measurements that mainly included cotinine and VOCs.

### HIV, HBV, and HCV diagnosis

The HIV-1 antibody status was determined by the blood test results of NHANES. According to the test results, the subjects were divided into two categories: HIV positive or HIV negative. The VITROS Hepatitis B surface antigen (HBsAg) test using by NHANES was used to determine the infection status of HBV, which was divided into HBV positive and HBV negative according to the test results. In addition, in vitro nucleic acid amplification test (COBAS AMPLICOR HCV MONITOR Test, version 2.0) was used to quantitatively determine HCV RNA in human serum or plasma to determine HCV infection status (HCV positive and HCV negative).

### Measurement of exposure and outcome

Participants' blood VOCs concentrations were automatically analyzed by capillary gas chromatography (GC) and mass spectrometry (MS) combined with selective ion monitoring (SIM) detection and isotope dilution. Serum cotinine was determined by isotope dilution high performance liquid chromatography / atmospheric pressure chemical ionization tandem mass spectrometry (ID HPLC-APCI-MS / MS). Smoking status was assessed from one self-reported item in the questionnaire: “Have you ever smoked cigarettes?”^25^ Thus, it might be described as “Smoker: yes/no” (as indicated in Supplementary Table S1), including smokers who reported they smoked at least 100 cigarettes in their lifetime and non-smokers who reported they had not smoked 100 cigarettes in their lifetime^26^. It was worth emphasizing that cotinine was defined as an effective biomarker of smoking in this study, while VOCs blood indicators were exposure biomarkers of cigarettes. We used the median (0.14 ng/mL) of serum cotinine in 3114 smokers as the boundary to divide it into a binary dependent variable (‘ ≥ 0.14 ng/mL’ was defined as high cotinine group of smokers; ‘ < 0.03 ng/mL’ was defined as low cotinine group of smokers), and the measured values of VOCs were included in the follow-up analysis as continuous independent variables. Similarly, the median (0.02 ng/mL) of serum cotinine in 10,538 non-smokers was defined as the boundary between high cotinine group (≥ 0.02 ng/mL) and low cotinine group (< 0.02 ng/mL) of non-smokers.

### Classification of confounders

Potential confounders in the relationship between VOCs and cotinine were selected for inclusion into multivariable logistic regression model. This study identified age, gender, race/ethnicity, education, family poverty index ratio (PIR), depression status, and substance use behavior. The sociodemographic variables included in this study were described in Supplementary Table S1. Only age (in years) was continuous variable, and the rest were classified variables.

### Statistical analysis

Independent Samples t-test was used for continuous variables that obeyed normal distribution, while Nonparametric test (Mann–Whitney U test) was used for continuous variables that did not obey normal distribution. All categorical variables were tested by Chi-square test. The single factor correlation analysis results were presented through the heat map based on Spearman algorithm. Correlation coefficient greater than 0.6 was defined as a strong correlation. Multivariable logistic regression analysis, adjusted for all potential confounders, was performed to determine the final relationship between VOCs and cotinine and to identify the independent risk factors leading to the increase of cotinine among smokers living with HIV, HBV, or HCV (HIV|HBV|HCV). The variance inflation factor was used to diagnose the multicollinearity between independent variables in the regression model (the multivariable regression model established in this study did not have multicollinearity, and all VIF values were less than 10). Cohen’s criteria for gauging small, medium and large effect sizes of R^2^ were 0.2, 0.5 and 0.8, which were calculated from Cohen’s d values^27^. A two-tailed *P* value less than 0.05 was considered statistically significant.

Data processing, statistical analysis, and graphic drawing were carried out with SAS version 9.4 (SAS Institute Inc., Cary, NC), IBM SPSS version 22.0, and R version 4.0.2 (http://www.R-project.org, The R Foundation).

### Institutional review board statement

Ethical review and approval were waived for this study, since all the data from NHANES is publicly accessible.

### Informed consent

Informed consent from all subjects was obtained by NHANES.

## Results

This study preliminarily compared the differences of sociodemographic variables, blood VOCs and cotinine between people living with HIV|HBV|HCV and those without HIV, HBV, and HCV (HIV&HBV&HCV). The sociological characteristics of the subjects and the test results of the differences of these blood indexes were shown in Supplementary Table S1. Compared with the HIV&HBV&HCV non-infected group, people living with HIV|HBV|HCV were more likely to be older (44.6 years vs 40.2 years, *P* < 0.001), males (67.4% vs 45.5%, *P* < 0.001), non-Hispanic Black (36.0% vs 19.3%, *P* < 0.001), lower educational level (45.0% vs 37.2%, *P* = 0.013), lower economic level (21.9% vs 15.7%, *P* = 0.010), depression (42.9% vs 32.9%, *P* = 0.009), drug users (60.3% vs 44.4%, *P* < 0.001), current smokers (44.6% vs 22.4%, *P* < 0.001). In addition, we also conducted a statistical test on the blood indexes between above two groups, and the results showed that the serum Cotinine (ng/mL), 1,2-Dichlorobenzene (ng/mL), Tetrachloroethene (ng/mL), Carbon Tetrachloride (ng/mL), Methylene Chloride (ng/mL), 1,1,1-Trichloroethane (ng/mL), and Nitrobenzene (ng/mL) were significantly (*P* < 0.05) higher in current smokers than in non-smokers.

**Figure 2:**
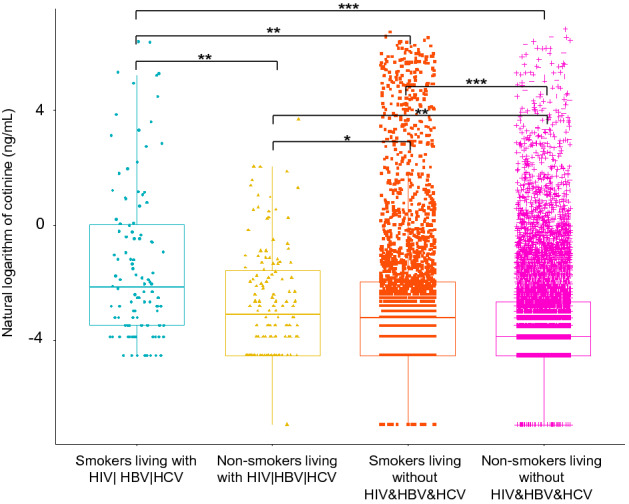
The distribution of serum cotinine levels in the different infection groups (***P < 0.001; **P < 0.01; *P < 0.05).

In order to further find possible differential serum VOCs, which might be associated with cotinine, we subdivided HIV|HBV|HCV infected group and HIV&HBV&HCV non-infected group into the following four subgroups (Supplementary Table S2): (1) Smokers living with HBV| HCV|HIV; (2) Non-smokers living with HIV|HBV|HCV; (3) Smokers living without HIV&HBV&HCV; (4) Non-smokers living without HIV&HBV&HCV. The results showed that the content of cotinine in the blood of the four groups was significantly different, that is, there was a statistical difference between each two groups (“Smokers living with HIV| HBV|HCV” *vs.* “Non-smokers living with HIV|HBV|HCV”, *P* < 0.01; “Smokers living with HIV| HBV|HCV” *vs.* “Smokers living without HIV&HBV&HCV”, *P* < 0.01; “Smokers living with HIV| HBV|HCV” *vs.* “Non-smokers living without HIV&HBV&HCV”, *P* < 0.001; “Non-smokers living with HIV|HBV|HCV” *vs.* “Smokers living without HIV&HBV&HCV”, *P* < 0.05; “Non-smokers living with HIV|HBV|HCV” *vs.* “Non-smokers living without HIV&HBV&HCV”, *P* < 0.05; “Smokers living without HIV&HBV&HCV” vs. “Non-smokers living without HIV&HBV&HCV”, *P* < 0.001). Figure 2 visualized above differences of serum cotinine content between each two groups. The results demonstrated that smokers living with HIV|HBV|HCV had significantly higher serum cotinine level than non-smokers living with HIV|HBV|HCV, and that smokers living without HIV&HBV&HCV had also significantly higher serum cotinine level than non-smokers living without HIV&HBV&HCV. Most obviously, smokers living with HIV|HBV|HCV had the highest serum cotinine level among the four groups. In addition, Supplementary Table S2 showed that four differential blood VOCs indicators, including 1,2-Dichlorobenzene (ng/mL), Tetrachloroethene (ng/mL), Benzene (ng/mL), and 2,5-Dimethylfuran (ng/mL), had positive dose effects on serum cotinine level.

**Figure 3:**
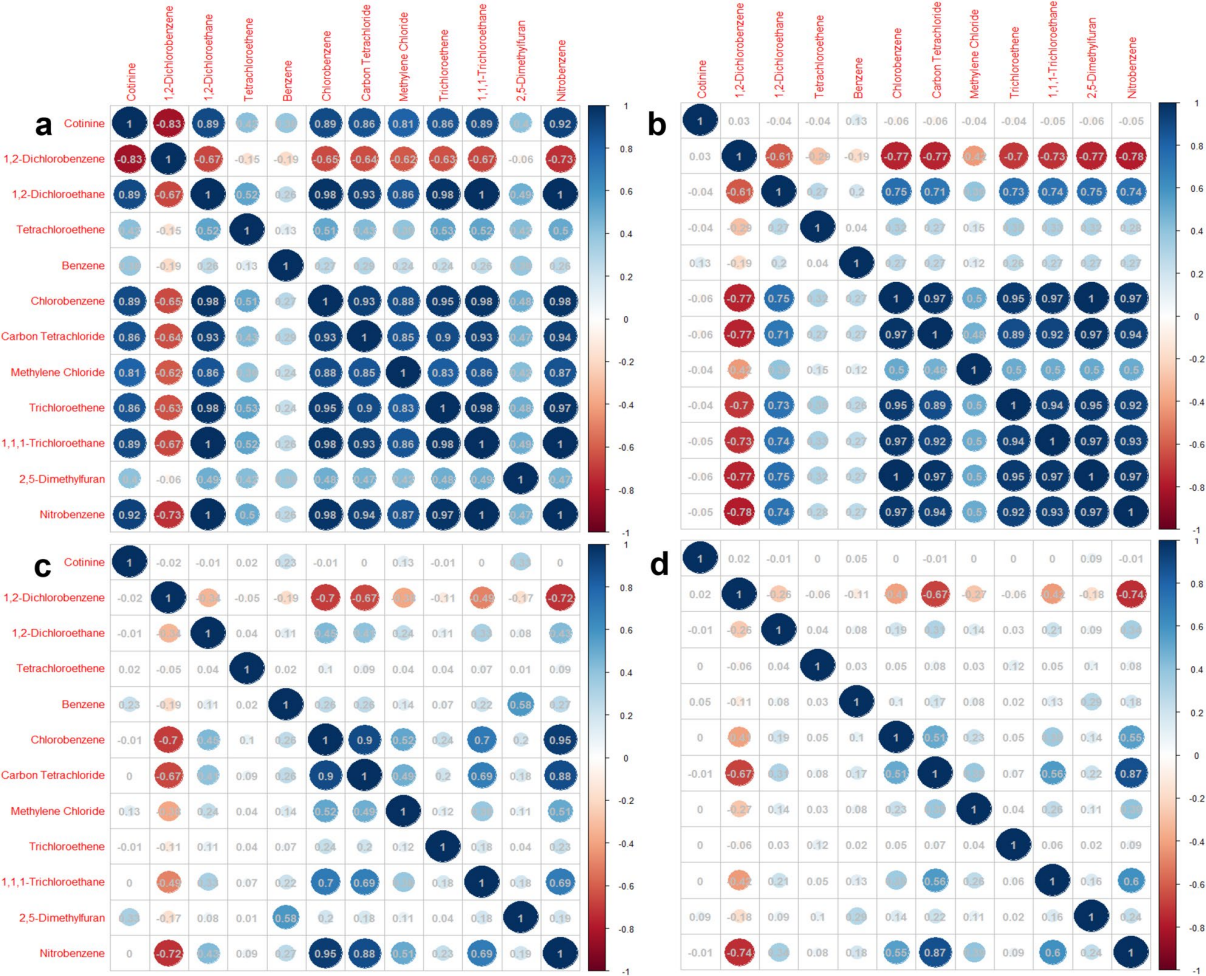
Spearman correlation analysis between serum VOCs and cotinine in different groups (**a.** Smokers living with HIV, HBV, or HCV; **b.** Non-smokers living with HIV, HBV, or HCV; **c.** Smokers living without HIV, HBV, and HCV; **d.** Non-smokers living without HIV, HBV, and HCV).

Subsequently, this study conducted Spearman correlation analysis on exploring the correlation between serum cotinine and VOCs among the above four groups. The results showed that the correlation heatmap between them was significantly different between different groups. From a macro perspective, the intensity and density of the correlation between blood indexes in the HIV|HBV|HCV infected group were higher than those in the HIV&HBV&HCV noninfected group. Compared to non-smokers living with HIV|HBV|HCV, the correlation between different blood indexes was more and stronger in smokers living with HIV|HBV|HCV. Compared to non-smokers living without HIV&HBV&HCV, the correlation between different blood indexes was more and stronger in smokers living without HIV&HBV&HCV. Specifically, there were 12 pairs of exclusively strong interaction items in smokers living with HBV|HCV|HIV (correlation coefficient greater than 0.6), including 1 pair of negative correlation indicators (correlation coefficient r = −0.83) and 12 pairs of positive correlation indicators (correlation coefficient fluctuation range is 0.81–0.92). All the above correlation coefficients mentioned were lower than the level of statistical significance (all *P* < 0.05), please see Fig. 3 for the details.

As for the association between serum VOCs and cotinine levels in 3114 smokers, multivariable binary logistic regression model, adjusting for age, gender, race/ethnicity, education, PIR, depression status, and drug use history, was conducted to identify potential associated VOCs of cotinine (Table 1). The results showed that 1,2-Dichlorobenzene, Benzene, Carbon Tetrachloride, 2,5-Dimethylfuran are all independent associated factors of cotinine. That was to say, high concentrations of 1,2-Dichlorobenzene (OR:1.036, CI:1.009–1.124), Benzene (OR:1.478, CI:1.036–2.292), Carbon Tetrachloride (OR:1.576, CI:1.275–2.085) and 2,5-Dimethylfuran (OR:1.091, CI:1.030–1.157) in blood might be independent risk factors leading to the increase of serum metabolite cotinine in smokers. Similarly, in 10,538 non-smokers, the results of the multivariable binary logistic regression model in Table 2 showed that high cotinine (≥ 0.02 ng/mL) group had higher levels of 1,2-Dichlorobenzene (OR:1.135, CI:1.079–1.193) and Carbon Tetrachloride (OR:1.493, CI:1.209–1.846), which might also be independent risk factors leading to the increase of serum metabolite cotinine in non-smokers.

**Table 1:** Association between serum VOCs and cotinine levels in 3114 smokers^†^.

Parameters	Low cotinine (< 0.14 ng/mL) group (Median and Percentiles (P_25_–P_75_))	High cotinine (≥ 0.14 ng/mL) group (Median and Percentiles (P_25_–P_75_))			95% Wald Confidence Interval for β			OR	95% Wald Confidence Interval for OR	
			β	Std. Error	Lower	Upper	*P* value		Lower	Upper
(Intercept)	–	–	− 1.412	0.526	− 2.442	− 0.381	0.007	0.244	0.087	0.683
1,2-Dichlorobenzene (ng/mL)	0.020 (0.010–0.048)	0.885 (0.280–20.650)	0.035	0.042	0.009	0.117	0.004	1.036	1.009	1.124
1,2-Dichloroethane (ng/mL)	0.050 (0.018–0.050)	0.050 (0.0177–0.050)	− 0.022	0.012	− 0.045	0.002	0.069	0.979	0.956	1.002
Tetrachloroethene (ng/mL)	0.005 (0.005–0.007)	0.005 (0.005–0.007)	0.011	0.240	− 0.459	0.481	0.964	1.011	0.632	1.617
Benzene (ng/mL)	0.012 (0.012–0.017)	0.016 (0.014–0.018)	0.397	0.221	0.036	0.830	0.027	1.487	1.036	2.292
Chlorobenzene (ng/mL)	0.006 (0.006–0.008)	0.006 (0.005–0.008)	− 0.007	0.018	− 0.042	0.028	0.677	0.993	0.958	1.028
Carbon Tetrachloride (ng/mL)	0.003 (0.003–0.004)	0.006 (0.002–0.007)	0.455	0.254	0.243	0.753	0.006	1.576	1.275	2.085
Methylene Chloride (ng/mL)	0.125 (0.125–0.177)	0.125 (0.125–0.177)	0.236	0.247	− 0.248	0.720	0.339	1.266	0.780	2.054
Trichloroethene (ng/mL)	0.006 (0.006–0.009)	0.008 (0.006–0.009)	− 0.285	1.222	− 2.681	2.111	0.816	0.752	0.069	8.254
1,1,1-Trichloroethane (ng/mL)	0.005 (0.005–0.007)	0.007 (0.005–0.007)	0.0003	0.0001	− 0.001	0.001	0.804	1.000	0.999	1.001
2,5-Dimethylfuran (ng/mL)	0.006 (0.006–0.008)	0.009 (0.005–0.009)	0.087	0.030	0.029	0.146	0.003	1.091	1.030	1.157
Nitrobenzene (ng/mL)	0.150 (0.159–0.226)	0.150 (0.150–0.226)	0.776	0.536	− 0.274	1.826	0.147	2.173	0.760	6.210

^**†**^Multivariable logistic regression analysis adjusted for age, gender, race/ethnicity, education, family poverty index ratio (PIR), depression status, and drug use history were adjusted. Std. Error, standard error; OR, odds ratio; N, sample size.

**Table 2:** Association between serum VOCs and cotinine levels in 10,538 non-smokers^†^.

Parameters	Low cotinine (< 0.02 ng/mL) group (Median and Percentiles (P_25_–P_75_))	High cotinine (≥ 0.02 ng/mL) group (Median and Percentiles (P_25_–P_75_))			95% Wald Confidence Interval for β			OR	95% Wald Confidence Interval for OR	
			β	Std. Error	Lower	Upper	*P* value		Lower	Upper
(Intercept)	–	–	− 1.000	0.315	− 1.617	− 0.382	0.002	0.368	0.198	0.683
1,2-Dichlorobenzene (ng/mL)	0.010 (0.010–0.010)	0.040 (0.020–0.130)	0.126	0.026	0.076	0.177	< 0.001	1.135	1.079	1.193
1,2-Dichloroethane (ng/mL)	0.050 (0.017–0.050)	0.050 (0.018–0.050)	0.0004	0.001	− 0.001	0.002	0.548	1.000	0.999	1.002
Tetrachloroethene (ng/mL)	0.005 (0.005–0.007)	0.005 (0.005–0.007)	− 0.227	0.153	− 0.527	0.073	0.139	0.797	0.591	1.076
Benzene (ng/mL)	0.012 (0.012–0.017)	0.012 (0.012–0.017)	0.153	0.114	− 0.070	0.376	0.180	1.165	0.932	1.456
Chlorobenzene (ng/mL)	0.006 (0.006–0.008)	0.006 (0.006–0.008)	0.002	0.003	− 0.003	0.007	0.477	1.002	0.997	1.007
Carbon Tetrachloride (ng/mL)	0.003 (0.002–0.004)	0.005 (0.002–0.006)	0.401	0.108	0.190	0.613	< 0.001	1.493	1.209	1.846
Methylene Chloride (ng/mL)	0.125 (0.125–0.177)	0.125 (0.125–0.177)	0.171	0.169	− 0.161	0.503	0.312	1.187	0.852	1.653
Trichloroethene (ng/mL)	0.006 (0.006–0.009)	0.006 (0.006–0.009)	− 0.006	0.155	− 0.309	0.297	0.970	0.994	0.734	1.346
1,1,1-Trichloroethane (ng/mL)	0.005 (0.005–0.007)	0.005 (0.005–0.007)	0.001	0.001	0.0001	0.002	0.113	1.001	1.000	1.002
2,5-Dimethylfuran (ng/mL)	0.006 (0.005–0.008)	0.006 (0.006–0.008)	0.076	0.046	− 0.015	0.166	0.101	1.079	0.985	1.181
Nitrobenzene (ng/mL)	0.150 (0.150–0.226)	0.150 (0.150–0.226)	0.250	0.256	− 0.252	0.751	0.329	1.283	0.777	2.119

^**†**^Multivariable logistic regression analysis adjusted for age, gender, race/ethnicity, education, family poverty index ratio (PIR), depression status, and drug use history. Std. Error, standard error; OR, odds ratio; N, sample size.

## Discussion

The harm of smoking to human health was well known, but few studies had mentioned the harmful effects of the synergistic factors (specifically VOCs in this study). Even though the studies of Pauwels C and others only studied the association between VOCs and smoking, they did not emphasize interactions or causal relationship between them^20,28,29^. Cotinine might be used as a biomarker of tobacco exposure for smokers because it was a main metabolite of nicotine (a compound whose concentration in the human body was produced in a high percentage by exposure to tobacco) and could be quantified in the body up to 72 h after the last cigarette^30^. However, Castellanos M believed that VOCs often accompanied by smoking were also the focus of studies, and could even be used as a biomarker of tobacco exposure^19^. It might be the reason that a large number of VOCs were produced in the process of smoking and competing with nicotine for cytochrome P450, which affected the metabolic efficiency of nicotine in the body, leading to smokers' addiction to tobacco and difficulty in quitting smoking^31,32^.

In this study, all subjects were divided into different groups (“People living with HBV, HCV, or HIV” and “People living without HBV, HCV, and HIV”), and the results showed that serum cotinine of smokers living with HBV, HCV, or HIV was the highest (Supplementary Table S1 and Fig. 2). In addition, we also found that all infected people had more and more dangerous sociological characteristics than non-infected people, which could explain that the distribution level of cotinine was actually affected by different socio-demographic characteristics. For example, compared with the HIV&HBV&HCV non-infected group, people living with HIV|HBV|HCV were more likely to be older, males, non-Hispanic Black, lower educational level, lower economic level, depression, drug users, current smokers. Consistently, one study also suggested that age, gender and race might affect the metabolism of nicotine and cotinine^33^. Therefore, this study adjusted above potential confounding factors, so as to obtain more accurate and reliable analysis results.

We also found a strong correlation between serum cotinine and eight VOCs (1,2-Dichlorobenzene, 1,2-Dichloroethane, Chlorobenzene, Carbon Tetrachloride, Methylene Chloride, Trichloroethene, 1,1,1-Trichloroethane, Nitrobenzene) in smokers infected with HIV|HBV|HCV. Studies showed that the smoking rate and tobacco exposure dose of this population were much higher than that of the general population^22,23,34,35^, so there were higher concentrations of cotinine and VOCs in the serum. Since the tobacco exposure of smokers infected with HIV|HBV|HCV was much higher than that of the general population, it could be inferred that the harm of serum cotinine and VOCs in the population would increase with the increase of tobacco exposure level. Our study also indicated a more obvious relationship between serum cotinine and VOCs in the population exposed to higher exposure of tobacco than lower the figure for tobacco. Therefore, we speculated that there might be a similar regularity in the non-infected population with the same unhealthy behavior (especially smoking in this study). In addition, there could be a possibility that the smoking cessation measures applied to the smokers of infected persons might also be extended to the non-infected smokers, which might indirectly provide new ideas for solving the public health problem of smoking. However, it requires more prospective multicenter cohort studies to prove whether this is possible in the future.

Our research also had some limitations. First of all, although the smokers among HIV|HBV|HCV infected people whose smoking rate was higher than that of the general population were integrated into the analysis, the sample size was still small. However, our study was consistent with other research results. For example, there was a significant positive correlation between serum cotinine and VOCs among smokers^18^. Secondly, the clear causal relationship between serum VOCs and cotinine needs to be further explored, because our study only found that the association between them in the body, and the relevant mechanism of serum cotinine level co-affecting by the external environment or other biochemical factors in the body might be more complicated than we expected. Finally, although NHANES had accurately measured the internal exposure dose of smoking, that exposure dose was far lower than the external exposure dose of subjects, so the existing data results might be underestimated.

## Conclusions

Among smokers living with HIV|HBV|HCV, the serum cotinine was the highest, and the association between serum VOCs and cotinine was also very significant. Therefore, there might be a dose–response relationship between the impact intensity of four VOCs (such as 1,2-Dichlorobenzene, Benzene, Carbon Tetrachloride, 2,5-Dimethylfuran) on serum cotinine metabolism, that is, the higher the tobacco exposure, the stronger the impact intensity. In addition, this study used molecular epidemiology to indirectly provide new ideas and perspectives on smoking hazards for smokers.

## Supplementary Information

Supplementary Information.

## Data Availability

Data described in the manuscript, codebook, and analytic code will not be made available because the data used in this study were from the NHANES database, which is a free and open database for all researchers around the world. The link to the database is https://wwwn.cdc.gov/nchs/nhanes/Default.aspx.
