# Supplementary material for: Association between serum cotinine and volatile organic compounds (VOCs) in adults living with HIV, HBV, or HCV (NHANES 2005–2018)
Source: Sci Rep. 2022 Dec 16;12:21769. doi: 10.1038/s41598-022-26420-7 (PMC9758166; doi:10.1038/s41598-022-26420-7)
Supplement: Supplementary file 1 — Supplementary Information. [file 41598_2022_26420_MOESM1_ESM.docx]

**Full Title:** Association between serum cotinine and volatile organic compounds (VOCs) in adults living with HIV, HBV, or HCV (NHANES 2005–2018)

**Authors: Jie Yang^1,#^, Hao Zhang^2, #^, Jin-Long Lin^3,4,#^, Jing Liu^5,#^, Xiao-Wen Jiang^6^, Lei Peng^7,*^**

^1^ Public Health Clinical Center of Chengdu, Chengdu 610066, China; yangjieHJ@gmail.com

^2^ Department of Social Medicine and Health Education, School of Public Health, Peking University, Beijing 100191, China; 2011210118@stu.pku.edu.cn

^3^ School of Marxism, Tsinghua University, Beijing 100084, China; jllin@pku.edu.cn

^4^ Institute of Population Research, Peking University, Beijing 100871, China; jllin@pku.edu.cn

^5^ People Liberation Army Haidian District 17th Retired Cadres Rest Home, Beijing 100143, China; musihuangwu131@163.com

^6^ Department of Epidemiology, School of Clinical Oncology, Peking University, Beijing 100142, China; jxw1911110605@pku.edu.cn

^7^ Department of Epidemiology, School of Clinical Oncology, Peking University, Beijing 100142, China

^#^ These authors contributed equally to this work.

^*^ Correspondence: 2011210593@stu.pku.edu.cn; Tel.: +86-138-0828-2849

**Supplementary table S1.** Demographic characteristics, serum indexes profile among people living with HIV|HBV|HCV compared to people without HIV&HBV&HCV (NHANES 2005–2018)

|  | **People living with HBV, HCV, or HIV (N=242)** | |  | **People living without HBV, HCV, and HIV (N=13,410)** | | **Effect size** | ***P* value ^†^** |
| --- | --- | --- | --- | --- | --- | --- | --- |
| **Characteristics** | **N** | **%** |  | **N** | **%** |  |  |
| Gender |  |  |  |  |  | 0.995 | <0.001 |
| Male | 163 | 67.4 |  | 6,101 | 45.5 |  |  |
| Female | 79 | 32.6 |  | 7,309 | 54.5 |  |  |
| Race/Ethnicity |  |  |  |  |  | 0.500 | <0.001 |
| Hispanic | 39 | 16.1 |  | 4,123 | 30.7 |  |  |
| Non-Hispanic White | 62 | 25.6 |  | 4,911 | 36.6 |  |  |
| Non-Hispanic Black | 87 | 36.0 |  | 2,588 | 19.3 |  |  |
| Other Race | 54 | 22.3 |  | 1,788 | 13.3 |  |  |
| Education |  |  |  |  |  | 0.906 | 0.013 |
| High school or less | 109 | 45.0 |  | 4,992 | 37.2 |  |  |
| Some college or more | 133 | 55.0 |  | 8,418 | 62.8 |  |  |
| Family poverty index ratio (PIR) |  |  |  |  |  | 0.892 | 0.010 |
| <Poverty level (PIR<1) | 53 | 21.9 |  | 2,106 | 15.7 |  |  |
| ≥Poverty level (PIR≥1) | 189 | 78.1 |  | 11,304 | 84.3 |  |  |
| Having depression **^a^** |  |  |  |  |  | 0.820 | 0.009 |
| Not at all | 88 | 57.1 |  | 5,815 | 67.1 |  |  |
| Yes (mild to severe depression) | 66 | 42.9 |  | 2,846 | 32.9 |  |  |
| Any drug user **^b^** |  |  |  |  |  | 0.985 | <0.001 |
| Yes | 140 | 60.3 |  | 5,837 | 44.4 |  |  |
| No | 92 | 39.7 |  | 7,321 | 55.6 |  |  |
| Alcohol use **^c^** |  |  |  |  |  | 0.680 | 0.331 |
| Yes | 26 | 10.7 |  | 1,199 | 8.9 |  |  |
| No | 216 | 89.3 |  | 12,211 | 91.1 |  |  |
| Smoker **^d^** |  |  |  |  |  | 0.994 | <0.001 |
| Yes | 108 | 44.6 |  | 3,006 | 22.4 |  |  |
| No | 134 | 55.4 |  | 10,404 | 77.6 |  |  |
|  |  | Median and Percentiles (P_25_–P_75_) |  |  | Median and Percentiles (P_25_–P_75_) |  |  |
| Age (years old) | — | 44.562 ± 10.086 |  | — | 40.215 ± 11.115 | 0.983 | <0.001 |
| Cotinine (ng/mL) | — | 0.070 (0.020–0.368) |  | — | 0.030 (0.010–0.080) | 0.141 | 0.042 |
| 1,2-Dichlorobenzene (ng/mL) | — | 0.050 (0.018–0.050) |  | — | 0.040 (0.017–0.051) | 0.673 | 0.025 |
| 1,2-Dichloroethane (ng/mL) | — | 0.007 (0.005–0.007) |  | — | 0.005 (0.005–0.007) | 0.071 | 0.659 |
| Tetrachloroethene (ng/mL) | — | 0.017 (0.012–0.017) |  | — | 0.012 (0.012–0.017) | 0.049 | 0.014 |
| Benzene (ng/mL) | — | 0.008 (0.006–0.008) |  | — | 0.006 (0.006–0.008) | 0.033 | 0.145 |
| Chlorobenzene (ng/mL) | — | 0.004 (0.003–0.004) |  | — | 0.003 (0.003–0.004) | 0.091 | 0.166 |
| Carbon Tetrachloride (ng/mL) | — | 0.155 (0.125–0.177) |  | — | 0.125 (0.120–0.168) | 0.502 | 0.037 |
| Methylene Chloride (ng/mL) | — | 0.008 (0.006–0.009) |  | — | 0.006 (0.006–0.009) | 0.637 | 0.022 |
| Trichloroethene (ng/mL) | — | 0.007 (0.005–0.007) |  | — | 0.005 (0.005–0.007) | 0.200 | 0.365 |
| 1,1,1-Trichloroethane (ng/mL) | — | 0.008 (0.006–0.008) |  | — | 0.006 (0.006–0.008) | 0.074 | 0.023 |
| 2,5-Dimethylfuran (ng/mL) | — | 0.212 (0.150–0.226) |  | — | 0.150 (0.150–0.226) | 0.055 | 0.072 |
| Nitrobenzene (ng/mL) | — | 0.070 (0.020–0.368) |  | — | 0.030 (0.010–0.080) | 0.063 | 0.025 |

^†^ Based on Chi-square test, Independent Samples t-test or Mann-Whitney U test between people with HIV|HBV|HCV and those without; ^a^ Based on having a score 1–4: no depression, and a score of 5–27: mild to severe depression in Patients Health Questionnaire (PHQ-9); ^b^ Ever use of any drug use including marijuana, hashish, cocaine, heroin, methamphetamine, and injection drugs; ^c^ 5 + (male) or 4 + (female) drinking every day at any time in the past; ^d^ smokers reported they smoked at least 100 cigarettes in their lifetime and non-smokers reported they have not smoked 100 cigarettes in their lifetime. N, sample size; SD, standard deviation.

**Supplementary table S2.** NHANES 2005–2018 US adults VOC blood level for current smokers and non-smokers based on infection status

| **Compounds** | **Smokers living with HBV\| HCV\|HIV**  **N=108** | **Non-smokers living with HBV\|HCV\|HIV**  **N=134** | **Smokers living without HBV&HCV&HIV**  **N=3,006** | **Non-smokers living without HBV&HCV&HIV**  **N=10,404** |
| --- | --- | --- | --- | --- |
| Cotinine (ng/mL) **^†^** | 0.115 (0.030–1.053) **^abc^** | 0.045 (0.010–0.215) **^de^** | 0.040 (0.010–0.140) **^f^** | 0.020 (0.010–0.070) |
| 1,2-Dichlorobenzene (ng/mL) **^†^** | 0.050 (0.018–0.050) | 0.034 (0.018–0.050) **^de^** | 0.050 (0.018–0.050) | 0.050 (0.018–0.050) |
| 1,2-Dichloroethane (ng/mL) **^†^** | 0.007 (0.005–0.007) | 0.007 (0.005–0.007) | 0.005 (0.005–0.007) | 0.005 (0.005–0.007) |
| Tetrachloroethene (ng/mL) **^†^** | 0.012 (0.012–0.017) **^bc^** | 0.017 (0.012–0.017) **^de^** | 0.012 (0.012–0.017) | 0.012 (0.012–0.017) |
| Benzene (ng/mL) **^†^** | 0.008 (0.006–0.008) | 0.008 (0.006–0.008) | 0.006 (0.006–0.008) **^f^** | 0.006 (0.006–0.008) |
| Chlorobenzene (ng/mL) **^†^** | 0.003 (0.003–0.004) | 0.004 (0.003–0.004) | 0.003 (0.003–0.004) | 0.003 (0.003–0.004) |
| Carbon Tetrachloride (ng/mL) **^†^** | 0.125 (0.125–0.177) | 0.177 (0.125–0.177) | 0.125 (0.125–0.177) | 0.125 (0.125–0.177) |
| Methylene Chloride (ng/mL) **^†^** | 0.008 (0.006–0.009) | 0.008 (0.006–0.009) | 0.006 (0.006–0.009) | 0.006 (0.006–0.009) |
| Trichloroethene (ng/mL) **^†^** | 0.007 (0.005–0.007) | 0.007 (0.005–0.007) | 0.005 (0.005–0.007) | 0.005 (0.005–0.007) |
| 1,1,1-Trichloroethane (ng/mL) **^†^** | 0.008 (0.006–0.008) | 0.008 (0.006–0.008) | 0.006 (0.006–0.008) | 0.006 (0.006–0.008) |
| 2,5-Dimethylfuran (ng/mL) **^†^** | 0.212 (0.150–0.226) | 0.212 (0.150–0.226) **^d^** | 0.150 (0.150–0.226) **^f^** | 0.150 (0.150–0.226) |
| Nitrobenzene (ng/mL) **^†^** | 0.115 (0.030–1.053) | 0.045 (0.010–0.215) | 0.040 (0.010–0.140) | 0.020 (0.010–0.070) |

**^†^** All of the above blood indexes are described in the form of Median and Percentiles (P_25_–P_75_) and tested by Mann-Whitney U test; **^a^** Compared with smokers living with HBV|HCV|HIV and non-smokers living with HBV|HCV|HIV, P < 0.05; **^b^** Compared with smokers living with HBV|HCV|HIV and smokers living without HBV&HCV&HIV, P < 0.05; **^c^** Compared with smokers living with HBV|HCV|HIV and non-smokers living without HBV&HCV&HIV, P < 0.05; **^d^** Compared with non-smokers living with HBV|HCV|HIV and smokers living without HBV&HCV&HIV, P < 0.05; **^e^** Compared with non-smokers living with HBV|HCV|HIV and non-smokers living without HBV&HCV&HIV, P < 0.05; **^f^** Compared with smokers living without HBV&HCV&HIV and non-smokers living without HBV&HCV&HIV, P < 0.05. N, sample size.
